# Histological analysis of autologous fascia graft implantation into the rabbit voice muscle

**DOI:** 10.1590/S1808-86942011000200008

**Published:** 2015-10-19

**Authors:** Bruno de Rezende Pinna, João Norberto Stavale, Paulo Augusto de Lima Pontes, Osíris de Oliveira Camponês do Brasil

**Affiliations:** 1 Medical doctor, master's degree student at the Otorhinolaryngology and Head & Neck Surgery Department, UNIFESP- EPM; 2 Habilitation title, Pathology Department, UNIFESP. Assistant professor, Pathology Department, UNIFESP; 3 Head of the Otorhinolaryngology and Head & Neck Surgery Department, UNIFESP- EPM. Full professor, Otorhinolaryngology and Head & Neck Surgery Department, UNIFESP- EPM; 4 Medical doctor, doctoral degree in science, Otorhinolaryngology and Head & Neck Surgery Department, UNIFESP- EPM. Universidade Federal de São Paulo - UNIFESP

**Keywords:** laryngeal diseases, glottis, fascia, rabbits, vocal cords

## Abstract

The ideal approach for the treatment of glottal insufficiency remains a challenge for laryngologists.

**Aim:**

This experimental study assessed the histological changes and fibrosis caused by autologous fascia lata grafts into the rabbit voice muscle.

**Study Design:**

A clinical and experimental study.

**Methods:**

A 0.2 × 0.2 cm fragment of autologous fascia lata was grafted into the right voice muscle of 14 adult rabbits. Animals were euthanized 30 or 60 days post-procedure and histology of the excised vocal folds was carried out.

**Results:**

No extensive edema, necrosis or foreign body-type reaction was observed at any time. No significant inflammatory reaction or fibrosis was seen at 30 or 60 days.

**Conclusion:**

The presence of fascia lata in the rabbit voice muscle had no significant influence on inflammation, and does not increase fibrosis. Rabbit voice muscle shows good tolerance to fascia lata grafting.

## INTRODUCTION

The main functions of the larynx are to protect lower airways and to produce voice. Glottic insufficiency due to incomplete contact between vocal folds may be caused by several glottal conditions, such as laryngeal palsy, scarring and sulcus vocalis.^1^ There are several methods for correcting glottic insufficiency, ranging from medialization techniques to injections of substances to increase vocal fold volume.^2^

Several points should be taken into account when choosing a biomaterial for implantation into the vocal fold. These include: ease of implantation, the type of immune response, and migration of the implanted material to undesired sites.^3^

Fascia grafts have been used for decades in ontological and facial plastic surgery.^4^ Its consistency is similar to that of collagen, and its metabolic activity is low. In 1998, Rihkanen introduced fascia lata for correcting glottic insufficiency.^5^ Reijonen et al.^6^ injected processed fascia lata into the vocal muscle of dogs in an experimental study, and found that the graft remained histologically identifiable one year later. On the other hand, Rodgers et al.^7^ described that processed autologous fascia injected into the vocal muscle was absorbed rapidly after three months.

Tsunoda et al.^8^ (2001) published several clinical trials in which autologous temporal fascia was implanted in the lamina propria and vocal muscle of human vocal folds. The authors concluded that temporal fascia was extremely compatible with Reinke's space. This was not the case if the temporal fascia was implanted in the vocal muscle. Nishiyama et al.^9^ (2006) observed excellent results with temporal fascia implants in the vocal muscle for patients with unilateral vocal fold palsy.

At the present time there is no consensus about which material is the best graft for the treatment of glottic insufficiency. Use of fascia lata has shown promising results, although few experimental studies have been carried out. These studies^6^, ^7^, ^10^ have observed the behavior of processed fascia lata injected into vocal folds; this procedure may be difficult, as it requires specific materials (Brunning's syringe) and is time-consuming. Tsunoda et al.^8^ has stated that the histopathological findings of fascia lata may be altered after processing, and that there is a higher risk of injected material being displaced to undesired sites. These authors suggested transplanting fascia into the vocal fold as a single block, under direct laryngoscopy.

We found no published paper assessing the histological behavior of a fascia lata fragment implanted into the vocal muscle by a cervical approach and not in contact with the respiratory mucosa.

## OBJETIVE

The purpose of this study was to analyze and compare the histological changes in autologous fascia lata fragments grafted into the vocal muscles of rabbits, 30 and 60 days after the procedure.

## METHODS

### Selection of animals

The sample consisted of 20 male New Zealand rabbits weighing from 1,950 to 2,550 grams; the animals were obtained from the Granja RG (RG Grange), which is located in the city of Suzano-SP. Rabbits were kept with adequate feeding and water supply at the bioterium of our institution. Vivisection of animals was done according to the norms in the Federal Law no. 6,638 of 8 May 1979 and the ethical principles of experimentation as define in the Brazilian Code of Animal Experimentation (COBEA).

### Surgical technique

Rabbits were anesthetized with xylazine (5 mg/ kg) and Zoletil^r^ (tiletamine chloridrate 125 mg/5 ml and zolazepam chloridrate 125 mg/5 ml); the dose was 0.4 mg/kg intramuscular. The animals were placed in dorsal decubitus on an operating table with a drainage channel. Orotracheal intubation was unnecessary.

Preoperative fur removal was done on the lateral portion of the right leg, followed by 10% PVP-I antisepsis, to harvest the fascia lata graft. The subcutaneous tissues and skin were closed with nylon 5-0 sutures. The next step was removal of fur and 10% PVP-I antisepsis of the anterior neck. An incision into the skin and subcutaneous tissue was made along the midline of the neck with a #15 blade scalpel. After exposing the thyroid cartilage, a #11 blade scalpel was used to open a 0.5 x 0.5 cm window in the right lamina; the upper border was left intact as a hinge for elevating the cartilage flap. After this dissection, the vocal muscle was identified and the fascia lata fragment was implanted. A delicate Kelly forceps was used in this procedure to make an intramuscular pouch for the 0.2 x 0.2 cm graft. Care was taken not to enter the pharyngeal lumen. The graft was implanted always on the right vocal fold. At the end of this procedure, the subcutaneous tissue and skin were closed with nylon 5.0 sutures.

The animals were given clindamycin 0.5 mg/kg intramuscular postoperatively for two days. Dipyrone 0.5 mg/kg was given for three days postoperatively. The animals were sacrificed by injecting 19.1% KCl (potassium chloride) 2ml and xylazine 1.5 mg/kg intracardiacally.

### Study groups

Subjects were allocated to two groups of 10 animals each. Group I consisted of animals that were euthanized after 30 days; group II consisted of animals that were euthanized after 60 days. The rabbits were numbered 1 to 20 and distributed randomly into groups by order of arrival to the bioterium and after an acclimatization period of at least 10 days. Following euthanasia, the larynx was fixed in 10% formaldehyde during 24 hours for histological studies at a later time. Upon removal of the larynx, the position and fixation of the cartilage window to its original site was noted.

Rabbits 6, 7 and 20 died between surgery and the fifth postoperative day, and were therefore excluded from the study.

### Histology

The examiner evaluated only the right vocal fold, without knowing to which group the animal belonged. Specimens were fixed in 10% formaldehyde during 24 hours. Coronal 5-micrometer sections across the membranous portion of the vocal fold were made. The specimens were hematoxylin-eosin (HE) and Masson's trichrome (TM) stained.

Histology was done with conventional microscopy to analyze the intensity of inflammation, predominant inflammatory cell type, necrosis, and feasibility of the fascia.

Tissues surrounding the implanted fascia were evaluated semiquantitatively according to its intensity on a weighted scale divided into four grades from absent to intense. Predominant cell types were also described, as follows: neutrophils (N), mast cells (M), lymphocytes (L), plasma cells (P), histiocytes (H), and eosinophils (E). Inflammation was graded as follows:

Absent (0): no inflammatory cells;

Mild (I): one to 10 cells were observed;

Moderate (II): 11 to 20 cells were observed;

Intense (III): over 20 cells were observed.

The intensity of scarring was assessed semiquantitatively. Surrounding tissues adjacent to the implanted fascia was compared with muscle beyond this area over three fields at X40 magnification. Scarring was graded as follows.

Absent (0): No collagen fibers;

Mild (I): sparse foci of collagen fibers were seen;

Moderate (II): continuous collagen bands around the graft;

Intense (III): long collagen fibers around the graft, forming a fibrous capsule.

These parameters were analyzed in the vocal fold around the grafted area and in the graft area. A comparison was made between rabbits in the 30-day group and the 60-day group. Fischer's exact test was applied for the statistical analysis of the variables.

## RESULTS

Of 20 rabbits that underwent surgery, three died after the procedure and before the fifth postoperative day. The fascia of rabbits 5, 12, and 14 was not found in the histological analysis; these animals were therefore excluded. Thus, each group effectively consisted of seven animals (Table 1).

**Table 1:** Number of animals according to the presence or absence of fascia in the histological analysis of both groups.

**Fascia**	**Group I**	**Group II**
Present	7	7
Absent	1	2

Total	8	9

Fisher's test (*p*)=1.0000

Not significant. A comparison of the absence of fascia showed no significant difference between both groups.

In rabbit number 14, the cartilage window was macroscopically seen to be loose within the larynx, which may explain why this graft was lost. Also, no hematoma was seen macroscopically.

Mild inflammation predominated in both groups (Table 2). Moderate scarring was seen in one case. Scarring was mild or absent in both groups (Table 3). There were no foreign body reactions (Table 4).

**Table 2:** Comparison between the right vocal fold (grafter) in group I and the right vocal fold (grafted) in group II: Comparative analysis of the intensity of inflammation in the grafted vocal folds in groups I and II

**Intensity of inflammation**	**GROUP I N / %**	**GROUP II N / %**
0	1 / 14.2	3 / 43
I	6 / 85.8	3 / 43
II	0 / 0	1 / 14.2
III	0 / 0	0 / 0

TOTAL	7 / 100	7 / 100

(0)- Absent; (I) Mild; (II) Moderate; (III) Intense

Fisher's test (*p*)=0.5594

Not significant. A comparison of the intensity of inflammation showed no significant difference between both groups.

Mild inflammation predominated in both groups.

**Table 3:** Comparative analysis of the intensity of scarring in the grafted vocal fold in groups I and II.

**Intensity of scarring**	**GROUP I N / %**	**GROUP II N / %**
0	2 / 29.0	1 / 14.2
I	4 / 56.8	6 / 85.2
II	1 / 14.2	0 / 0
III	0 / 0	0 / 0

TOTAL	7 / 100	7 / 100

(0)- Absent; (I) Mild; (II) Moderate; (III) Intense

Fisher's test (*p*)=1.0000

Not significant. A comparison of the intensity of scarring showed no significant difference. Mild scarring predominated.

**Table 4:** Comparative analysis of the presence of foreign body chronic reaction among grafted vocal folds in both groups.

**Presence of giant cell reaction**	**GROUP I N / %**	**GROUP II N / %**
0	7 / 100	7 / 100
I	0 / 0	0 / 0

TOTAL	7 / 100	7 / 100

(0) Absent; (I) Present

Fisher's test (*p*)=1.0000

Not significant. A comparison of the presence of foreign body chronic reaction among grafted vocal folds showed no significant difference in both groups.

No foreign body reaction was seen in any of the cases.

## DISCUSSION

Fascia consists of a single cell type (fibroblasts) and an intercellular collagen matrix; both are found in the lamina propria of vocal folds. In theory, fascia implanted as an autologous graft is not expected to generate an immune response; it is expected to be incorporated into the graft site.

We found moderate scarring in one of our cases. These small areas of scarring may have resulted mostly from the surgical procedure itself than to the presence of the graft; this finding differs from the results presented by Reijonen et al.^6^ and Rodgers et al.^7^ These different results may be explained by the fact that processed fascia contains more fibroblast fragmentation, which may have caused scarring.

A comparison of the intensity of inflammation in both groups showed that it was either absent or mild; it was moderate in one case only. Inflammation was mostly absent in group II, but the difference was not statistically significant. Inflammation in group I (30 days) may have been due mostly to surgery.

**Figure 1:**
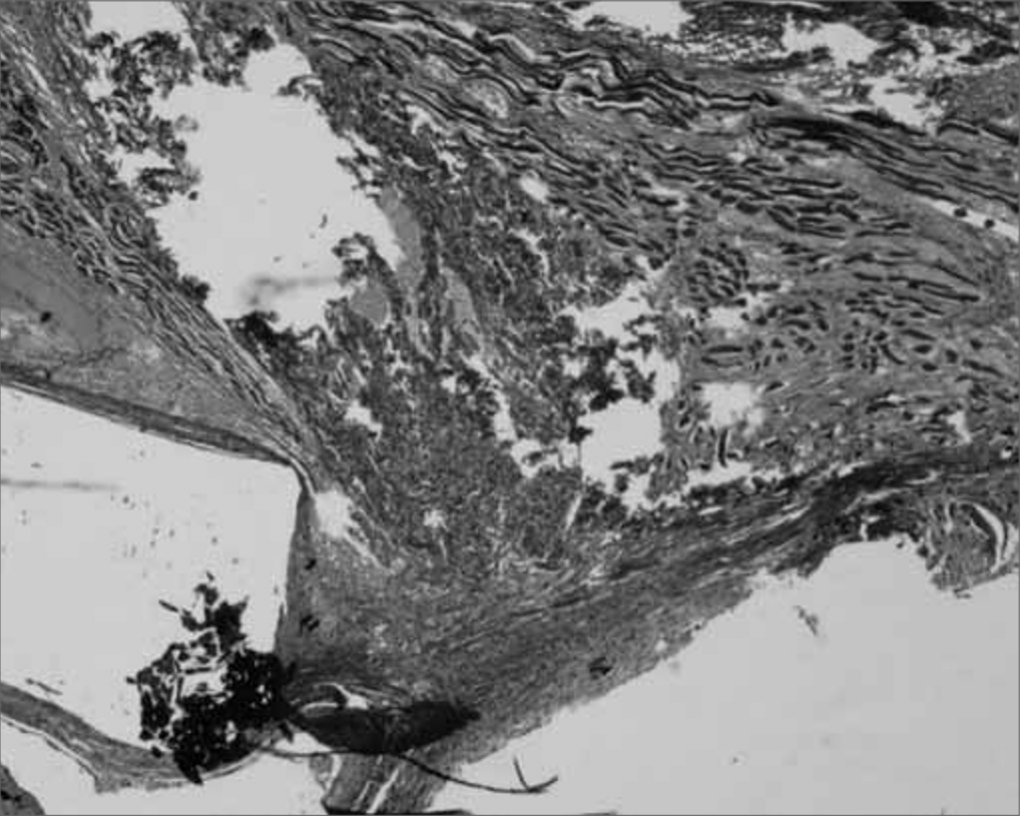
Masson's trichrome. 40x - right vocal fold. Grafted right vocal fold with fascia. Note the graft in blue within the vocal muscle, in a rabbit euthanized after 60 days. There is no inflammation.

**Figure 2:**
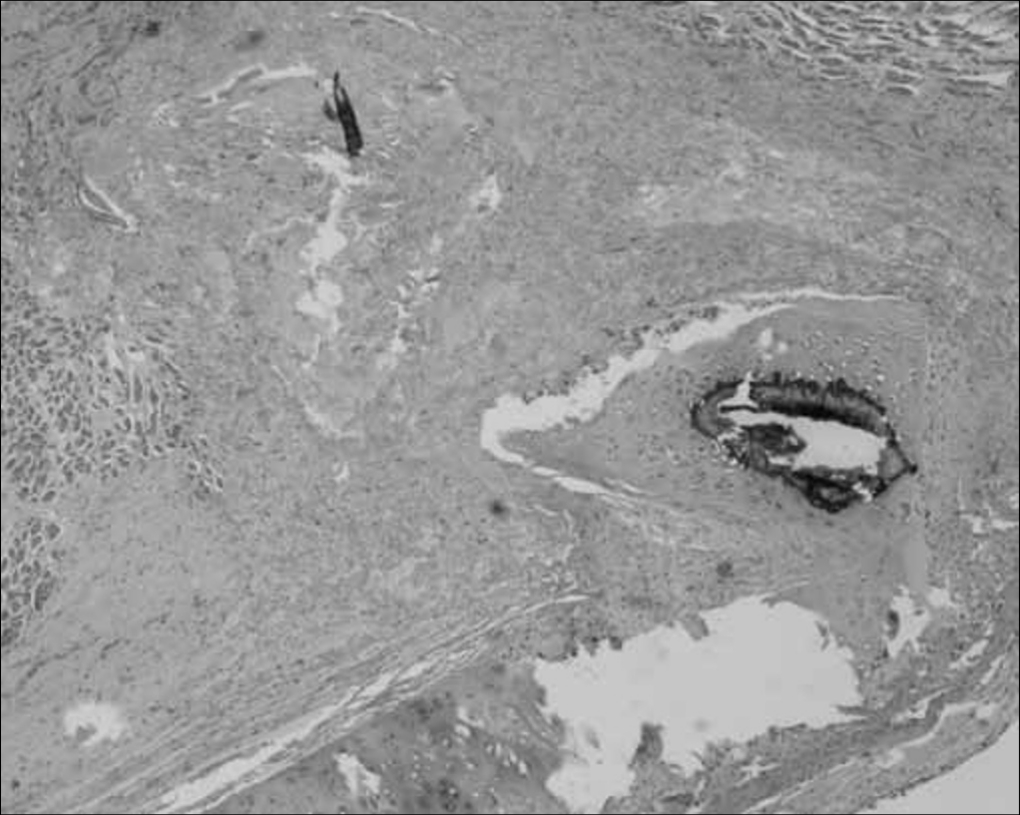
Hematoxylin-eosin. 40X - right vocal fold. Fascia graft in the vocal muscle of a rabbit euthanized after 60 days. Note mild scarring in the vocal muscle. The implanted fascia may be seen.

We did not investigate the absorption rate of fascia. The graft was not found in three cases; we therefore chose to exclude these cases from the study, since it could not be established whether the fascia had been absorbed or extruded. Reijonen et al.^6^ found injected fascia lata grafts up to 12 months later in the vocal fold of dogs. Thus, we do not believe that fascia was absorbed after 60 days in our study. In one case the window was not fixed to the larynx (rabbit 14, group II), which may explain graft loss in this case. Tsunoda et al.^11^ and Nishiyama et al.^12^ described wound closure with absorbable 7.0 sutures in the direct laryngoscopy techniques; the purpose was to avoid the implanted material from extruding. These authors found that the sutures disappeared after three months in their follow-up. We consider that fixation of the cartilage window should be considered to improve the technique we used.

Absence of activated macrophages in histology is another finding that demonstrated biocompatibility between fascia and the vocal fold. Macrophages coalesce and become activated when any substance is recognized as foreign to tissues; it is then phagocytosed and eliminated. The presence of activated macrophages characterizes foreign body chronic inflammation, a finding that we did not see in our samples.^13^

Reijonen et al.^6^ (2001) found that the volume of injected fascia decreased after 12 months in the vocal muscle of dogs. We did not study the absorption rate of fascia; when it was found, the fascia appeared intact.

Rodgers et al.^7^ (2000) found unfavorable results when injecting fascia lata in the vocal muscle of dogs. After three months, the fascia could no longer be recognized histopathologically. Their conclusion was that fascia lata was not a good material to be injected in the vocal muscle.

Reijonen's histological results showed that fascia is well tolerated when injected in the vocal fold muscle of dogs. This authors found no granulomas or necrosis at any time during the study, and concluded that fascia was a safe graft;^6^ our findings were similar. Graft-induced inflammation was mild in the 30-day and the 60-day groups. There was no necrosis or foreign body reaction in both groups.

Tsunoda et al.^8^ (2001) suggested that fascia implants in the lamina propria would induce similar tissue regenerating results to those of stem cells. These authors found that patients submitted to fascia implants in the lamina propria had better results than those undergoing fascia implants in the vocal muscle. Nishiyama^9^ (2006) found excellent results after implanting fascia in the vocal muscle. We did not compare inflammation induced by fascia lata in the vocal muscle and in the lamina propria.

Several approaches to the larynx in experimental rabbit models have been published. Duprat^14^ used laryngofissure to expose both vocal folds in rabbits. Other authors, such as Hertegard et al.^15^, used zero degree optics for children. Campognolo et al.^16^ proposed using a laryngoscope based on Killian's nasal speculum. We carried out procedures in 26 rabbits where both vocal folds were well exposed in all animals. The authors reported an easy and fast technique with few complications.

We chose a technique that would provide easy handling and a low contamination risk of the vocal muscle in rabbits. Approaching the paraglottic space and the vocal muscle through a window in the cartilage lamina is a well-established technique since 1915.^17^ It provides excellent exposure of the vocal fold on the side of the procedure; exposure of the contralateral vocal fold is difficult. In this study we analyzed only one vocal fold. In future studies using the same technique, it may be necessary to generate improvements for adequate exposure of both vocal folds. In 20 operated rabbits, only one cartilage window was seen to be loose macroscopically, relative to the laryngeal structure (rabbit 14, group II).

The planned sample was 20 rabbits. We believe that excluding 6 animals (30%) may have significantly reduced our sample.

## CONCLUSION

We concluded in this study that fascia lata, used as an autologous graft, caused little inflammation and scarring in the vocal muscle of rabbits.
